# Validación del reactivo fCAL turbo Buhlmann para la determinación de calprotectina en suero

**DOI:** 10.1515/almed-2025-0174

**Published:** 2025-11-12

**Authors:** Helena Čičak, Željka Šarčević, Ana Sruk, Frane Bukvić, Daria Pašalić, Lora Dukić, Ana-Maria Šimundić

**Affiliations:** Department of Medical Laboratory Diagnostics, University Hospital “Sveti Duh”, Zagreb, Croacia; Department of Medical Laboratory Diagnostics, University Hospital “Sveti Duh”, Zagreb, Croacia; Department of Neurology, University Hospital “Sveti Duh”, Zagreb, Croacia; Department of Orthopaedic Surgery and Traumatology, University Hospital “Sveti Duh”, Zagreb, Croacia; Department of Medical Chemistry, Biochemistry and Clinical Chemistry, University of Zagreb School of Medicine, Zagreb, Croacia; Faculty of Pharmacy and Biochemistry, University of Zagreb, Zagreb, Croacia

**Keywords:** calprotectina, preanaítico, suero, verificación

## Abstract

**Objetivos:**

El objetivo del presente estudio es evaluar la validez del reactivo fCAL turbo, que se emplea para la determinación de la calprotectina en muestras fecales, para las muestras séricas.

**Métodos:**

Se utilizaron muestras de suero sobrantes obtenidas de los pacientes. El protocolo de validación incluyó un estudio de precisión realizado conforme a las directrices EP15-A3 del Clinical and Laboratory Standards Institute (CLSI); un análisis comparativo entre los reactivos Bühlmann fCAL turbo y Gentian GCAL; la verificación de los intervalos de referencia (IR) propuestos en la literatura (0,68–5,45 mg/L), siguiendo las recomendaciones de la guía EP28-A3C del CLSI; la estimación del límite de blanco (LB) y del límite de cuantificación (LQ) según la guía EP17-A2 del CLSI; y la evaluación de la linealidad conforme a la guía EP6-A. También se comprobó el arrastre y del efecto ”hook”. Los criterios de aceptación se establecieron en función de las especificaciones del fabricante incluidas en el manual de uso del reactivo fCAL turbo Buhlmann para el análisis de la calprotectina en muestras fecales.

**Resultados:**

Los coeficientes de variación (CV) obtenidos en el estudio de precisión cumplieron los criterios de aceptación establecidos, con valores≤10 %. El análisis Bland-Altman reveló la presencia de un sesgo de 3,4 mg/L (intervalo de confianza del 95 %. 2,00–4,79). La ecuación para la regresión de Passing-Bablok fue y= −0,01 (−0,08 – 0,06) + 0,37 (0,36–0,40)x, mostrando una diferencia proporcional. El LB obtenido fue de 0 mg/L, con un LQ de 0,04 mg/L (CV – 20 %.). El método fue lineal en el intervalo 0,04–4,14 mg/L, no habiendo detectado arrastre (0,1 %.) o efecto hook. Validamos los IR propuestos en la literatura empleando 20 muestras sobrantes de pacientes.

**Conclusiones:**

Aunque, según el fabricante, el reactivo Buhlmann fCAL turbo está destinado únicamente al análisis de muestras fecales, también se puede emplear para la determinación de la calprotectina en suero. Por otro lado, el reactivo Buhlmann fCAL turbo no es intercambiable con el reactivo GCAL Gentian.

## Introducción

Un número creciente de publicaciones científicas pone de relieve el valor potencial de la calprotectina en suero como biomarcador en múltiples contextos patológicos, incluyendo la gastroenterología, la ortopedia y otros campos de la medicina. Las mayores concentraciones de calprotectina se encuentran a nivel celular, en particular en los neutrófilos, que son la primera línea de defensa contra los patógenos y el tipo de leucocito más numeroso en sangre. Durante la primera respuesta inmunitaria, los neutrófilos y otros inmunocitos liberan calprotectina en el ambiente donde se produce la reacción del sistema inmune, normalmente en el tejido adyacente o en los fluidos corporales (FC) [1].

La cuantificación de la concentración de calprotectina en FC como el líquido sinovial puede aportar información de relevancia clínica sobre el nivel de inflamación local. Sin embargo, no existe un protocolo estandarizado para la toma de muestras de FC, que en ocasiones no se puede repetir cuando el volumen de la muestra resulta insuficiente. Además, la sangre liberada durante la punción traumática puede causar interferencia y provocar la contaminación de la muestra de FC. En estos casos, la concentración experimental de calprotectina en el FC será incorrecta, no porque la sangre aporte un gran volumen de calprotectina, sino porque se desconoce, o no se puede calcular, su aportación exacta de calprotectina. Así mismo, en algunos casos, realizar un seguimiento de las concentraciones de calprotectina podría ser de utilidad en el proceso de toma de decisiones clínicas sobre el paciente. Desafortunadamente, no se suelen tomar muestras sucesivas de FC, ya que ello implica la utilización de técnicas invasivas y entraña el riesgo de que se produzcan complicaciones asociadas al proceso de recogida de la muestra.

Por otro lado, sí existe un protocolo estandarizado para la extracción de muestras de sangre venosa, un procedimiento que se puede repetir tantas veces como sea preciso. Además, el procedimiento para la extracción de muestras de suero o plasma es más sencillo que el empleado para obtener muestras de FC. Hasta la fecha, no se han llevado a cabo estudios para validar el empleo del reactivo fCAL turbo Buhlmann, cuyo uso está muy extendido en el análisis de muestras fecales para la cuantificación de la calprotectina en suero. Por ejemplo, se desconoce el límite de cuantificación de este analito.

Hasta hace poco, la determinación de la calprotectina fecal únicamente se podía realizar empleando los kits y reactivos ELISA para analizadores automáticos, desarrollados tras validarse la aplicación clínica de este analito y ser este introducido en los análisis sistemáticos. En los últimos años, se han diseñado reactivos para cuantificar la calprotectina en suero (listos para usar) empleando analizadores automáticos. Sin embargo, existen solo unos pocos estudios sobre la validación de la calprotectina en suero o plasma, en los cuales se emplearon los kits de reactivos fCAL turbo (Buhlmann Laboratories AG, Schonenbuch, Suiza), GCAL-Gentian Serum Calprotectin (Gentian Diagnostics, Moss, Noruega), el ensayo DiaSorin Liaison Calprotectin (DiaSorin, Saluggia, Italia), y el ensayo inmunoenzimático fluorescente EliA Phadia 250 para la cuantificación de la calprotectina [2], [3], [4], [5]. Hasta donde sabemos, no se han llevado a cabo grandes estudios destinados a la validación o verificación de reactivos para determinar la concentración de calprotectina en muestras de suero.

En nuestro estudio, planteamos la hipótesis de que el reactivo para la determinación de la calprotectina fecal también sería válido para cuantificar la concentración de calprotectina en muestras séricas. El objetivo del presente estudio es evaluar la validez del reactivo Buhlmann fCAL turbo calculando su precisión, realizando un análisis comparativo entre este reactivo y GCAL Gentian, y validando a su vez los intervalos de referencia propuestos en la literatura científica. Finalmente, determinamos el límite de blanco, el límite de cuantificación, la linealidad, el arrastre y el efecto hook.

## Materiales y métodos

En el estudio, llevado a cabo en el Hospital Universitario Sveti Duh de Zagreb (Croacia), empleamos muestras séricas sobrantes tras realizar las pruebas analíticas solicitadas a los pacientes. Se introdujeron las muestras en tubos de 4 mL con activador de coágulo (REF 11010) (Vacutest Kima s.r.l., Arzergrande, Italia). Las muestras de sangre venosa se recogieron siguiendo las recomendaciones de la Federación Europea de Química Clínica y Medicina de Laboratorio (EFLM) y la Confederación Latinoamericana de Bioquímica Clínica (COLABIOCLI) [6].

Una vez recogidas y enviadas al laboratorio, las muestras se centrifugaron en una centrífuga VWR MEGA STAR 1.6 (VWR International GmbH, Vienna, Austria) a 4,000 rpm durante 10 minutos. Posteriormente, se realizaron las pruebas solicitadas por los facultativos, en las dos horas siguientes a la recogida de la muestra. Inmediatamente después, utilizamos las muestras de suero sobrantes para realizar los análisis de nuestro estudio. Para ello, empleamos el reactivo Buhlmann fCAL turbo (Buhlmann Labora-tories AG, Schonenbuch, Suiza) para cuantificar las concentraciones de calprotectina en un analizador Atellica Solution (Siemens, Erlangen, Alemania) con la configuración recomendada por el fabricante. Cabe señalar que este reactivo está destinado a la determinación de la calprotectina en heces. Previamente al análisis, las muestras fecales se diluyen en un tampón de extracción a una proporción de 1:500. Para la realización del análisis, no es necesaria la extracción y dilución de las muestras séricas. Debido a la dilución, los resultados obtenidos se dividieron entre 500, por lo que todos los objetivos de verificación proporcionados por el fabricante, así como los resultados obtenidos con el analizador se expresaron en.ig/g, indicando entre paréntesis las concentraciones resultantes de la división. Además, según el fabricante, se puede realizar la conversión de los resultados de.ig/g a mg/L aplicando un factor de 1,0. Los dos reactivos evaluados (fCAL turbo Buhlmann y Gentian) se fundamentan en la misma metodología analítica: la inmunoturbidimetría. La muestra se mezcla con inmunopartículas recubiertas de anticuerpos específicos de la calprotectina. La calprotectina presente en la muestra reacciona con las inmunopartículas generando una aglutinación que aumenta la turbidez; esta se mide mediante la absorbencia de la luz, que es proporcional a la concentración de calprotectina en la muestra. Para el análisis del reactivo fCAL turbo Buhlmann, empleamos los calibradores y controles B-KCAL-CONSET (lote 3616) y B-KCA-CASET (lote 3616), mientras que para el reactivo Gentian utilizamos el kit de calibración GCAL (lote 2007406) y el kit de control GCAL (2007409). El estudio se llevó a cabo con la aprobación del Comité de Ética del hospital para la utilización de muestras sanguíneas sobrantes.

### Evaluación de la precisión

Para estimar la precisión, se utilizaron dos muestras de pacientes. El estudio de precisión se llevó a cabo siguiendo las directrices EP15-A3 del CLSI [7]. Analizamos dos muestras con diferentes concentraciones de calprotectina [alta (A) y baja (B)] cinco veces durante cinco días consecutivos. Las muestras se repartieron en cinco alícuotas y se almacenaron a −20 °C para su posterior análisis. El criterio de aceptación que figuraba en las instrucciones de uso de Buhlmann fCAL turbo fue un CV del 10 %.

### Comparación de métodos

Para analizar las muestras séricas de 40 pacientes, empleamos dos reactivos distintos: a) Buhlmann fCAL turbo y b) GCAL – Gentian Serum Calprotectin (Gentian Diagnostics, Moss, Noruega). Todas las muestras se midieron en una sola tanda y se analizaron en un sistema Atellica Solution de Siemens. Tal como se comentó anteriormente, los resultados obtenidos con el reactivo Buhlmann fCAL turbo se dividieron por 500. Los resultados de Gentian no precisaban el empleo de coeficientes de conversión.

### Validación del límite de blanco y el límite de cuantificación

El límite de blanco (LB) y el límite de cuantificación (LC) se estimaron siguiendo la guía EP17-A2 del CLSI [8]. Analizamos dos muestras de solución salina durante tres días consecutivos con las siguientes réplicas diarias: 3-3-4 (20 réplicas en total). El LB declarado por Buhlmann fue de 16,7 µ/g (0,03 mg/L). El criterio de aceptación para la verificación del LB es del 85 %, esto es, 17 de cada 20 resultados debían ser iguales o inferiores al LB indicado por el fabricante.

Para estimar el LQ, empleamos muestras con una concentración baja de calprotectina. La dilución de muestras se realizó con un diluyente CH (Siemens, Erlangen, Alemania), tal como recomienda el fabricante, con el fin de obtener concentraciones bajas de calprotectina (cercanas al LQ indicado por el fabricante). Analizamos las muestras durante tres días consecutivos realizando las siguientes réplicas diarias: 3-3-4 al día (20 réplicas en total). En la documentación técnica del analizador Atellica Solution de Siemens, el fabricante declara un LQ de 20 μg/g (0,04 mg/L), mientras que en las especificaciones generales del reactivo figura un LQ de 23,7 μg/g (0,05 mg/L) [9], 10]. Se comprobó el LQ declarado para el analizador que empleamos en nuestro estudio. El criterio de aceptación para la verificación del LQ fue un CV del 20 %.

### Validación de la linealidad

La linealidad indicada por el fabricante fue de 20–2000 iµ/g (0,04–4,00 mg/L). El análisis de linealidad se llevó a cabo siguiendo la guía EP6-A del CLSI [11]. Empleamos dos muestras de pacientes, una con una concentración alta (A) próxima al extremo superior de la linealidad declarada, y otra con una concentración baja (B) cercana al extremo inferior. La muestra con una concentración alta de calprotectina se obtuvo agregándole calprotectina recombinante humana (n° de referencia HC2120, HycultBiotech Inc., Wayne, EE.UU). Se realizaron diluciones seriadas para generar seis rangos de concentración, obtenidos al combinar las muestras baja (B) y alta (A) en proporciones de volumen predeterminadas, según se detalla a continuación: 1) B, 2) 0,8B+ 0,2A, 3) 0,6B+ 0,4A, 4) 0,4B+ 0,6A, 5) 0,2B+ 0,8A, and 6) A. Las muestras se analizaron una única vez. A continuación, comparamos los resultados con los valores teóricos, que se calcularon tras determinar las concentraciones A y B. El criterio de aceptación para el sesgo máximo fue un CV inferior al 10 %, tal como indica el fabricante [7].

### Evaluación del arrastre

Repartimos dos muestras séricas, una con una concentración de calprotectina alta (A) y otra baja (B), en cuatro alícuotas cada una, que se analizaron en el siguiente orden: A1, A2, A3, A4, B1, B2, B3, y B4. Seguidamente, calculamos el arrastre aplicando la siguiente fórmula: {B1 -(B3 + B4)/2}/{(A3 + A2)/2 – (B3 + B4)/2} *100, y se consideró aceptable si se obtenía un valor<1 %. [12].

### Efecto ”hook”

El fabricante no indica la existencia de efecto hook. La muestra elegida se enriqueció con calprotectina recombinante humana (n° ref. HC2120, HycultBiotech Inc., Wayne, EE.UU). Tras determinar su concentración de calprotectina, se obtuvieron nueve muestras diluidas mediante diluciones seriadas con un diluyente CH. Si la concentración de las muestras diluidas superaba la de la muestra inicial enriquecida, se confirmaba la presencia de efecto gancho [3].

### Validación de los intervalos de referencia (IR)

Según establece la guía EP28-A3C del CLSI, para verificar el intervalo de referencia de la calprotectina, se deben incluir 20 muestras de suero sobrantes de pacientes que se sometieron a un examen médico general [13]. En esta parte del estudio, únicamente se incluyó a pacientes sin patologías previas o existentes. Los criterios de exclusión fueron; a) presencia de patologías o enfermedades inflamatorias agudas o crónicas (la PCR, la sedimentación de eritrocitos y el valor absoluto de leucocitos debían encontrarse en los límites de normalidad); b) pacientes de entre 18 y 65 años; y c) muestras hemolizadas o lipémicas. Aunque el fabricante no proporciona intervalos de referencia para las muestras séricas, los estudios publicados indican un intervalo de referencia de 0,68–5,45 mg/L para la concentración de calprotectina obtenida con el reactivo Buhlmann fCAL turbo en muestras séricas [2]. Los criterios de aceptación para verificar los IR es que 18 de los 20 resultados obtenidos se encuentren dentro del IR propuesto.

### Análisis estadístico

El análisis estadístico de los datos obtenidos se realizó con Microsoft 365 Excel (Microsoft, Washington, EE.UU), empleando estadística descriptiva para la caracterización de los resultados. Los datos que no seguían una distribución normal se presentaron como medias y rangos intercuartílicos (RIC). Asimismo, para evaluar la equivalencia de los dos métodos, se realizó un análisis estadístico con el diagrama de Bland-Altman y la regresión de Passing-Bablok, empleando el software estadístico MedCalc versión 20.027 (Ostende, Bélgica). Como nivel de significación estadística, se estableció un valor de p<0,05.

## Resultados

### Validación de la precisión

Para validar la precisión de los dos métodos, se empleó una concentración de 2,29 mg/L en la muestra 1 y 3,94 mg/L en la muestra 2 de los pacientes. Con respecto a los CV (coeficientes de variación) entre las dos muestras, la variabilidad intraserial fue del 2,22 %., siendo la variabilidad interserial del 6,73 %., y la variabilidad dentro de un mismo laboratorio del 7,09 %. en la muestra 1, frente al 1,19 %., 8,16 %., y 8,25 %. en la muestra 2. Todos los CV se encontraban dentro del criterio de aceptación establecido.

### Comparación de los métodos

**Figura 1:**
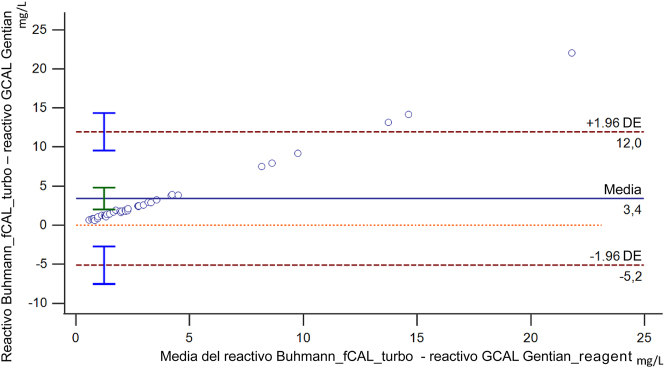
Diagrama de Bland-Altman para la comparación de métodos muestra un sesgo absoluto al comparar el reactivo de calprotectina Buhlmann fCAL turbo con GCAL-Gentian. La línea azul continua representa el sesgo absoluto, con el intervalo de confianza (IC) del 95 %. señalado con una barra verde de error.

En las Figuras 1 y 2 se muestra la comparación de métodos para la determinación de la calprotectina en suero. El diagrama de Bland-Altman analysis reveló una desviación constante positiva de 3,4 (IC 95 %. 2,00–4,79) y una desviación proporcional positiva del 92,8 %. (IC 95 %. 89,95.–95,56 %.). La prueba de suma acumulada de la regresión de Passing-Bablok no mostró ninguna desviación significativa de la linealidad (p=0,150). La ecuación de regresión fue y= −0,01 (entre −0,08 y 0,06) + 0,37 (0,36–0,40)x, mostrando una diferencia proporcional.

**Figura 2:**
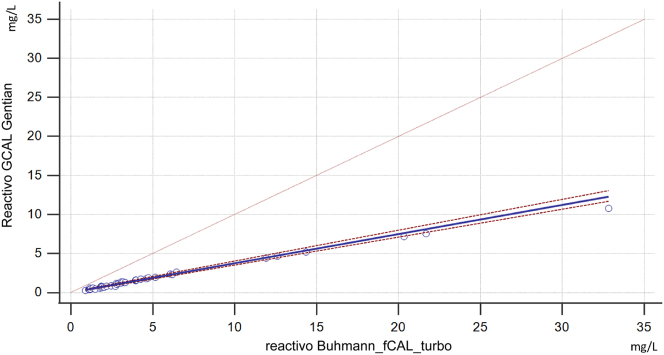
Regresión de Passing-Bablok de dos determinaciones empleando los reactivos Buhlmann fCAL turbo y GCAL – Gentian. En el eje de abscisas se muestran las determinaciones obtenidas con el reactivo Buhlmann, mientras que en el eje de coordenadas se indican las obtenidas con el reactivo de Gentian. La línea continua azul es una línea de regresión con el IC del 95 %, que se representa en la línea discontinua roja.

### Validación del límite de blanco y el límite de cuantificación

La totalidad de los resultados (20 de 20) del análisis de las dos muestras salinas fueron inferiores a los LB indicados por el fabricante. Validamos el LB de 16,7 µ/g (0,03 mg/L). Así mismo, en todas las muestras salinas se obtuvo un resultado de 0 mg/L.

En el análisis de las dos muestras de suero de dos pacientes diferentes, las concentraciones fueron de 20,5 µ/g (0,04 mg/L) y 22,1 (0,04 mg/L) µg/g, con unos CV de 13,7 %. y 12,1 %., respectivamente. Los CV cumplían el criterio de aceptación (≤20 %.), y comprobamos el LQ indicado por el fabricante (20,0 µ/g (0,04 mg/L)).

### Validación de la linealidad

La concentración de calprotectina de la muestra que contenía una concentración baja fue de 20,82 µ/g (0,04 mg/L), frente a los 2070,21 µ/g (4,14 mg/L) de la muestra con una concentración alta. Calculamos las concentraciones teóricas de las diluciones predeterminadas. Los sesgos de los valores teóricos y experimentales fueron inferiores al 10 %, salvo en la dilución de 0,8B+ 0,2A, donde el sesgo fue del 10,5 %. En la Tabla 1 se muestran los datos de linealidad.

**Tabla 1:** Datos obtenidos en la validación de la linealidad.

Valor del nivel de concentración	Dilución	Concentración teórica, µg/g (mg/L)	Concentración experimental, µg/g (mg/L)	Sesgo absoluto	Sesgo relativo
1	L	20,82 (0,04)	20,82 (0,04)	0	0
2	0,8 L+0,2 H	430,70 (0,86)	476,05 (0,95)	0,11	10,5
3	0,6 L+0,4 H	840,58 (1,68)	923,59 (1,85)	0,10	9,90
4	0,4 L+0,6 H	1250,46 (2,50)	1285,04 (2,57)	0,03	2,80
5	0,2 L+0,8 H	1660,33 (3,32)	1634,24 (3,27)	−0,02	−1,60
6	H	2070,21 (4,14)	2070,21 (4,14)	0	0

Muestra B con una concentración baja de calprotectina. Muestra A con una concentración alta de calprotectina.

### Evaluación del arrastre

La concentración media de calprotectina fue de 1.62486 µ/g (3,25 mg/L) en la muestra A y 42,65 µ/g (0,09 mg/L) en la muestra B. El arrastre experimental fue de 0,1 %, lo que no cumple el criterio de aceptación predeterminado de<1 %.

### Efecto ”hook”

**Figura 3:**
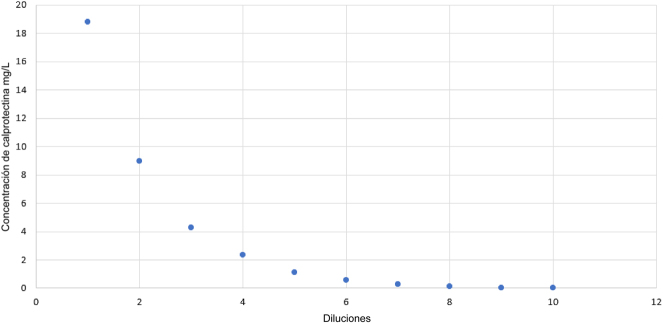
Representación gráfica del efecto “hook”. En el eje de coordenadas está representada la concentración de calprotectina, mientras que en el eje de abscisas se muestra el número de diluciones de la muestra.

La concentración experimental de la muestra enriquecida fue de 9,413.51 μg/g (18,83 mg/L). El nivel más alto de calibrador empleado, el kit de calibradores fCAL turbo (Buhlmann Laboratories AG, Schonenbuch, Suiza) (lote 2915) fue de 2285,7 μg/g. Ninguno de los resultados de la dilución seriada de la muestra enriquecida fue superior al resultado de la muestra enriquecida. No observamos efecto hook a la hora de determinar la concentración de calprotectina en las muestras séricas (Figura 3).

### Validación de los intervalos de referencia (IR)

En el presente estudio, empleamos 20 muestras sobrantes de suero pertenecientes a pacientes ambulatorios que cumplieron los criterios de inclusión. La edad media de los pacientes fue de 45 años, siendo la edad mínima 34 años y la máxima 57 años. La mayoría de los pacientes incluidos eran mujeres (15/20). Se validó el IR propuesto en la literatura científica (0,68–5,45 mg/L), dado que 20 de los 20 resultados se encontraban dentro de dicho IR.

## Discusión

Nuestro estudio de validación demuestra una precisión, LB, LQ y linealidad aceptables, evidenciando la ausencia de efecto hook y arrastre. Sin embargo, uno de cada seis niveles de concentración de linealidad superaba el criterio predeterminado de solo el 0,5 % o, en términos de sesgo absoluto, 0,11 mg/L, que no es clínicamente significativo. Además, se validó el IR propuesto en la literatura científica para la calprotectina en suero obtenida con el reactivo fCAL turbo Buhlmann. Detectamos un sesgo proporcional significativo al comparar el reactivo fCAL turbo Buhlmann con el reactivo GCAL Gentian.

Por un lado, los resultados obtenidos coinciden con algunos estudios publicados. Por ejemplo, Asberg A et al. también emplearon el reactivo Buhlmann fCAL turbo para cuantificar la calprotectina en plasma, demostrando que el método era lineal, con un CV experimental para la precisión de<4 %., el cual es similar al obtenido en nuestro estudio [2]. Por otro lado, nuestros hallazgos no se ven respaldados por los resultados de otros estudios. Nilsen T y col [3]. compararon el reactivo Gentian GCAL con el kit de reactivos Buhlmann MRP8/14 ELISA, elaborado por el mismo fabricante que uno de los reactivos empleados en nuestro estudio (fCAL turbo). Desafortunadamente, la regresión de Passing-Bablok se mostraba sin el IC 95 % correspondiente, por lo que resulta imposible determinar la presencia de un sesgo constante o proporcional. Por lo tanto, no podemos determinar si su hallazgo concuerda con nuestros resultados. Cabe señalar que Nilsen T. y col [3]. concluyeron que el inmunoensayo de Gentian para la calprotectina era equivalente y mostraba una buena correlación con el método Buhlmann MRP8/14 ELISA, a pesar de que estos reactivos se fundamentan en metodologías distintas. Sin embargo, nuestros resultados indican que los reactivos GCAL Gentian y Buhlamnn fCAL turbo no son equivalentes debido a la presencia de un sesgo proporcional. Aunque los hallazgos sean similares, la discrepancia entre los resultados de todos estos estudios se podría deber a diferencias metodológicas en las pruebas empleadas.

Una de las limitaciones de este estudio es que se podría haber incluido un número mayor de muestras para que la comparación entre métodos hubiera presentado un rango más amplio de concentraciones. Así mismo, se debería investigar la linealidad con medidas por duplicado. Aunque Asberg y col [2]. determinaron que no existen diferencias en el IR en función del sexo o la edad, otra limitación del presente estudio en la desproporción entre la participación femenina y masculina a la hora de verificar los IR. Además, también se podrían haber verificado los IR con el reactivo GCAL Gentian. Por último, la presencia de arrastre se podría volver a investigar con otras y más numerosas muestras de suero. Finalmente, se podría plantear realizar en el futuro un estudio de validación más amplio en el que se incluyeran más muestras y algunos de los reactivos introducidos recientemente en el mercado.

Cabe mencionar que los valores de los IR verificados fueron superiores a la linealidad indicada por el fabricante para el reactivo empleado en este estudio, lo que podría ser de utilidad para el desarrollo de nuevos reactivos de calprotectina en suero. Además, sería deseable investigar la calidad analítica del nuevo reactivo de calprotectina sérica presentado por el fabricante Buhlmann, cuyo reactivo fCAL turbo empleamos en este estudio. Por otro lado, sería conveniente realizar estudios para validar los distintos métodos de determinación de la calprotectina, ya que no existen patrones internacionales, lo que lleva a los fabricantes a emplear patrones internos distintos y obtener resultados no coincidentes.

Aunque ya se ha investigado con éxito el papel de la calprotectina sérica como nuevo biomarcador para el diagnóstico y seguimiento de ciertas patologías, y de haberse investigado su uso y relevancia clínica, aún es necesario validar los reactivos actualmente existentes para la cuantificación de la calprotectina. El desarrollo de nuevos reactivos para la determinación de la concentración de calprotectina debería ir acompañado de su validación por parte del personal de laboratorio previamente a su integración en la práctica, con el fin de garantizar la obtención de resultados fiables y exactos en los informes de laboratorio.

## Conclusiones

En conclusión, el estudio de validación de la precisión, LB, LQ, linealidad, arrastre y efecto gancho del reactivo de Buhlmann coincide con la mayoría de los valores declarados por el fabricante en sus especificaciones, así como con los criterios predeterminados para la calprotectina en suero, a pesar de haber sido diseñado para la cuantificación de la calprotectina en muestras de heces humanas. El reactivo fCAL turbo Buhlmann es válido para el análisis de la concentración de calprotectina en muestras séricas. Por otro lado, el reactivo fCAL turbo Buhlmann no es equivalente al reactivo GCAL Gentian, no siendo por lo tanto intercambiables.
